# Identification of Differentially Expressed Genes and Pathways in Non-Diabetic CKD and Diabetic CKD by Integrated Human Transcriptomic Bioinformatics Analysis

**DOI:** 10.3390/ijms26157421

**Published:** 2025-08-01

**Authors:** Clara Barrios, Marta Riera, Eva Rodríguez, Eva Márquez, Jimena del Risco, Melissa Pilco, Jorge Huesca, Ariadna González, Claudia Martyn, Jordi Pujol, Anna Buxeda, Marta Crespo

**Affiliations:** 1 Department of Nephrology, Hospital del Mar, 08003 Barcelona, Spain; erodriguezg@hmar.ca (E.R.); eva.marquez.mosquera@hmar.cat (E.M.); jimena.delrisco.zevallos@hmar.cat (J.d.R.); melissalorena.pilco.teran@hmar.cat (M.P.); jorge.huesca.campillo@hmar.cat (J.H.); ariadna.gonzalez.garcia@hmar.cat (A.G.); abuxeda@hmar.cat (A.B.); mcrespo@hmar.cat (M.C.); 2 Hospital del Mar Research Institute, 08003 Barcelona, Spain; cmartyn@researchmar.net (C.M.); jordipb2002@gmail.com (J.P.); 3 RICORS2040-Renal, 08003 Barcelona, Spain

**Keywords:** non-diabetic chronic kidney disease, diabetic chronic kidney disease, transcriptomics, differential expression analysis, gene set enrichment analysis

## Abstract

Chronic kidney disease (CKD) is a heterogeneous condition with various etiologies, including type 2 diabetes mellitus (T2D), hypertension, and autoimmune disorders. Both diabetic CKD (CKD_T2D) and non-diabetic CKD (CKD_nonT2D) share overlapping clinical features, but understanding the molecular mechanisms underlying each subtype and distinguishing diabetic from non-diabetic forms remain poorly defined. To identify differentially expressed genes (DEGs) and enriched biological pathways between CKD_T2D and CKD_nonT2D cohorts, including autoimmune (CKD_nonT2D_AI) and hypertensive (CKD_nonT2D_HT) subtypes, through integrative transcriptomic analysis. Publicly available gene expression datasets from human glomerular and tubulointerstitial kidney tissues were curated and analyzed from GEO and ArrayExpress. Differential expression analysis and Gene Set Enrichment Analysis (GSEA) were conducted to assess cohort-specific molecular signatures. A considerable overlap in DEGs was observed between CKD_T2D and CKD_nonT2D, with CKD_T2D exhibiting more extensive gene expression changes. Hypertensive-CKD shared greater transcriptomic similarity with CKD_T2D than autoimmune-CKD. Key DEGs involved in fibrosis, inflammation, and complement activation—including *Tgfb1*, *Timp1*, *Cxcl6*, and *C1qa/B*—were differentially regulated in diabetic samples, where GSEA revealed immune pathway enrichment in glomeruli and metabolic pathway enrichment in tubulointerstitium. The transcriptomic landscape of CKD_T2D reveals stronger immune and metabolic dysregulation compared to non-diabetic CKD. These findings suggest divergent pathological mechanisms and support the need for tailored therapeutic approaches.

## 1. Introduction

Chronic kidney disease (CKD) represents a global health burden, affecting more than 10% of the population worldwide [1]. It is a progressive disorder and a major contributor to morbidity and mortality, which contributes to accelerated aging and all-cause and mainly cardiovascular premature death [2,3]. CKD is a complex syndrome that encompasses and reflects the activation of numerous pathophysiological pathways that are in many cases common, regardless of their etiology. Both genomic and environmental factors contribute to this complex heterogeneous disease [4]. CKD heritability is estimated to be high (30–75%) [5,6], and there is a strong association between an individual’s genetic make-up and their predisposition to develop nephropathy. For example, it has been shown that up to 35% of the patients with diabetes develop nephropathy, irrespective of glycemic control [7]. Genome-wide association studies (GWAS) and GWAS meta-analyses have proposed several genetic loci associated with CKD [8,9]. However, these genetic markers do not account for all the susceptibility to CKD, and the causal pathways remain incompletely understood. Among the various etiologies, type 2 diabetes mellitus (T2D) is the leading cause of CKD, with diabetic nephropathy accounting for a substantial proportion of patients progressing to end-stage renal disease. Nevertheless, CKD also arises in non-diabetic settings, often driven by hypertension or autoimmune glomerulopathies, and regardless of overlapping clinical outcomes, the pathophysiological mechanisms driving diabetic and non-diabetic CKD may diverge significantly.

Despite their clinical relevance, comparative molecular profiling between diabetic and non-diabetic CKD forms has remained limited. Integrating high-throughput gene expression and transcriptomics analysis facilitates a better understanding of disease mechanisms by revealing changes in gene expression across tissues. However, few studies have comprehensively contrasted the molecular landscapes of CKD_T2D and CKD_nonT2D—including its autoimmune and hypertensive subtypes—at the level of individual genes and biological pathways.

This study aims to bridge that gap by integrating and analyzing publicly available transcriptomic datasets of kidney tissues, focusing on glomeruli and tubulointerstitium, to elucidate shared and distinct molecular signatures between CKD_T2D and CKD_nonT2D. Through differential expression analysis (DEGs) and gene set enrichment (GSEA), we provide insights into tissue-specific processes underpinning disease progression and uncover potential molecular targets for precision medicine in CKD.

## 2. Results

### 2.1. Dataset Compilation and Selection

**Figure 1:**
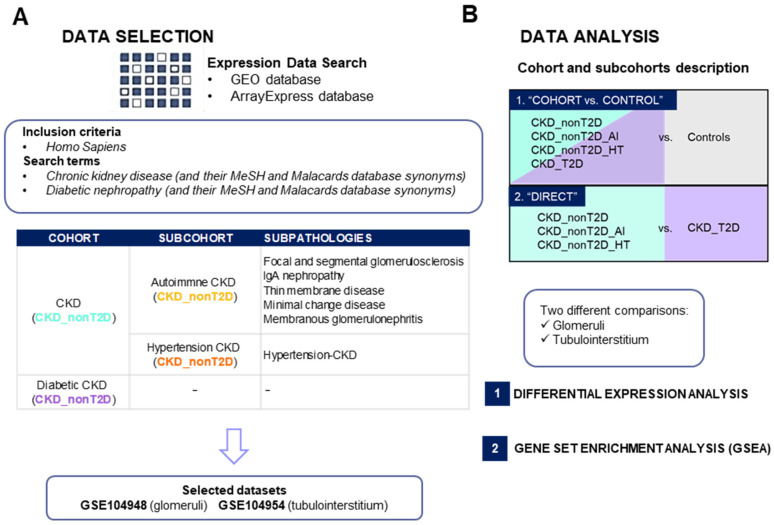
Study summary of dataset selection (**A**) and gene expression analysis (**B**) in chronic kidney disease. AI: Autoimmune; CKD_T2D: chronic kidney disease cohort associated with type 2 diabetes mellitus; CKD_nonT2D: chronic kidney disease cohort not associated with type 2 diabetes; GEO: Gene Expression Omnibus; HT: hypertensive. To aid interpretation, different colors were used to distinguish study cohorts: ice blue for CKD_nonT2D, mauve for CKD_nonT2D, topaz for CKD_nonT2D, and tangerine for CKD_nonT2D.

From an initial pool of 174 datasets retrieved from GEO (*n* = 95) and ArrayExpress (*n* = 79), 30 met the inclusion criteria (GEO (*n* = 18) and ArrayExpress (*n* = 12)) (Figure 1). After removing duplicates, 20 datasets were included. Of these, only experiments containing both cohorts of interest (CKD_nonT2D and CKD_T2D), as well as healthy controls, were prioritized in front of studies containing only one of the two conditions of interest (CKD_T2D or CKD_nonT2D, either autoimmune or hypertensive), obtaining two relevant datasets, GSE104954 (tubulointerstitium) and GSE104948 (glomeruli). Samples of the two relevant datasets clustered according to tissue, as illustrated in Supplementary Figure S1. Figure 1A shows how the datasets were obtained and selected. For the other kidney datasets two (GSE175759 and GSE30529) contained healthy controls and a cohort of interest. GSE30529 was obtained following the same experimental procedure as the primary analysis datasets, microarray, while GSE175759 samples were analyzed through RNAseq. Therefore, only GSE30529 was used to validate the results obtained from the primary analysis.

### 2.2. Differential Expression Analysis Results

#### 2.2.1. Chronic Kidney Disease Glomeruli and Tubulointerstitium Transcriptomic Profile Differences Between Cohorts

Differential expression analysis (DEA) was performed to identify differentially expressed genes (DEGs) between diabetic or non-diabetic CKD populations against controls and directly between CKD_T2D patients and those with CKD_nonT2D and its sub-cohorts, as schematized in Figure 1B. A complete list of the differentially expressed genes is shown in the Supplementary Material (Supplementary Table S1), which compiles all the information found for each gene during the analysis for the different comparisons performed. Log fold change (logFC) and Adj. *p*-value scores are provided for both the “CKD-cohort vs. control” and direct comparison “CKD_T2D vs. CKD_nonT2D” both in glomeruli and tubulointerstitium.

**Figure 2:**
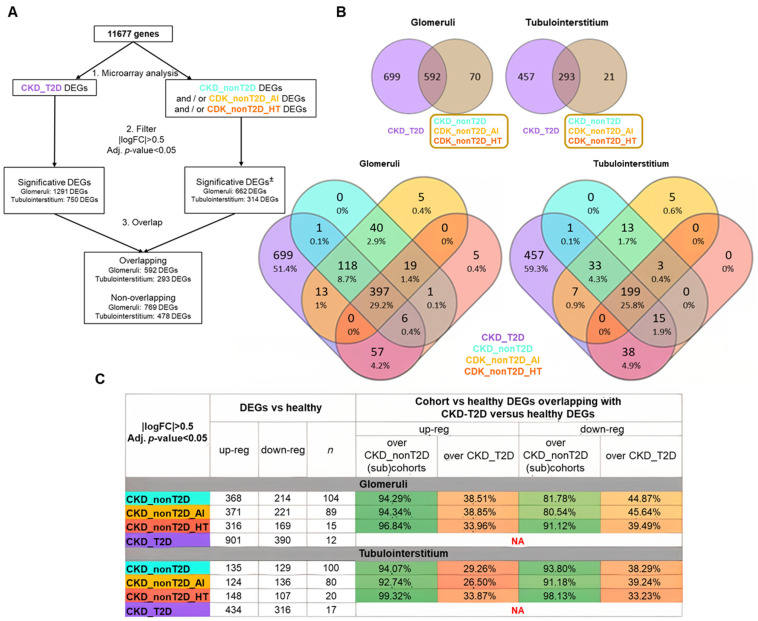
DEGs from comparing cohorts/subcohorts against healthy control samples. (**A**) Description of the path followed during the analysis. (±) Significative DEGs must satisfy the filter for, at least, CKD_nonT2D or one of its subcohorts, but not for all of them. Overlapping DEGs are defined as those that show an overlap between CKD_T2D and CKD_nonT2D or any of its subcohorts. (**B**) Venn diagrams. CKD_nonT2D and CKD-T2D overlap for glomeruli and tubulointerstitium. (lower panels) CKD_nonT2D, CKD-T2D, CKD_nonT2D_AI, and CKD_nonT2D_HT overlap for glomeruli and tubulointerstitium. (**C**) Summary of the differential expression analysis in glomeruli and tubulointerstitium and the proportion of DEGs in common between diabetic and non-diabetic cohorts compared to healthy, *n* = number of samples. DEGs: differentially expressed genes; AI: Autoimmune; CKD_T2D: chronic kidney disease cohort associated with type 2 diabetes mellitus; CKD_nonT2D: chronic kidney disease cohort not associated with type 2 diabetes; HT: hypertensive. To aid interpretation, different colors were used to distinguish study cohorts: ice blue for CKD_nonT2D, mauve for CKD_nonT2D, topaz for CKD_nonT2D, and tangerine for CKD_nonT2D.

Both glomeruli and tubulointerstitium samples were analyzed independently. The results of this comparison were filtered according to the pre-established thresholds (adjusted *p*-value < 0.05; |logFC| > 0.5), with genes fulfilling these conditions being considered DEGs (Figure 2).

Subsequently, the DEGs against healthy control of the CKD_T2D cohort were compared with the significant DEGs of the CKD_nonT2D cohort and its subcohorts. Two categories of DEGs were established: (1) overlapping DEGs, as those that presented overlap between the CKD_T2D and CKD_nonT2D or any of its subcohorts; (2) non-overlapping DEGs, such as those DEGs that did not present overlap between those of CKD_T2D and those of CKD_nonT2D or any of its subcohorts. No DEGs were observed to be significantly increased in the CKD_T2D cohort and decreased in CKD_nonT2D, and vice versa: all the significant overlapping DEGs followed the same direction.

In order to ascertain the degree of similarity between cohorts, the proportion of DEGs in common between diabetic and non-diabetic cohorts compared to healthy ones was examined. Figure 2C summarizes these findings, and, for example, in glomeruli 347 increased DEGs are shared between CKD_NonT2D and CKD_T2D, representing 94.29% of all increased DEGs in CKD_NonT2D (368) and 38.51% of all increased DEGs in CKD_T2D (901). Both glomeruli and tubulointerstitium increased (or decreased) genes always represent more than 80% when comparing with non-diabetic cohorts; meanwhile, when comparing over the diabetic cohort, these genes represent 26.5–45.64%. Therefore, while the majority of the changes from the non-diabetic cohort are also present in the diabetic cohort, in this last cohort more than half of the DEGs are not present in the first cohort, suggesting that kidneys from the diabetic populations also have other changes not present in the non-diabetic cohort.

The GSE30529 dataset (containing 10 samples from CKD_T2D and 12 samples from living donors tubulointerstitium biopsies) was used to validate the results obtained in GSE104954 in the CKD-T2D versus control comparison. This analysis showed that 95% of DEGs in both studies maintain the same regulation trend, and only 5% show reverse trends (Supplementary Figure S2).

#### 2.2.2. Similarities and Differences of Diabetic CKD Transcriptomic Profile with Non-Diabetic Cohort/Subcohorts

**Figure 3:**
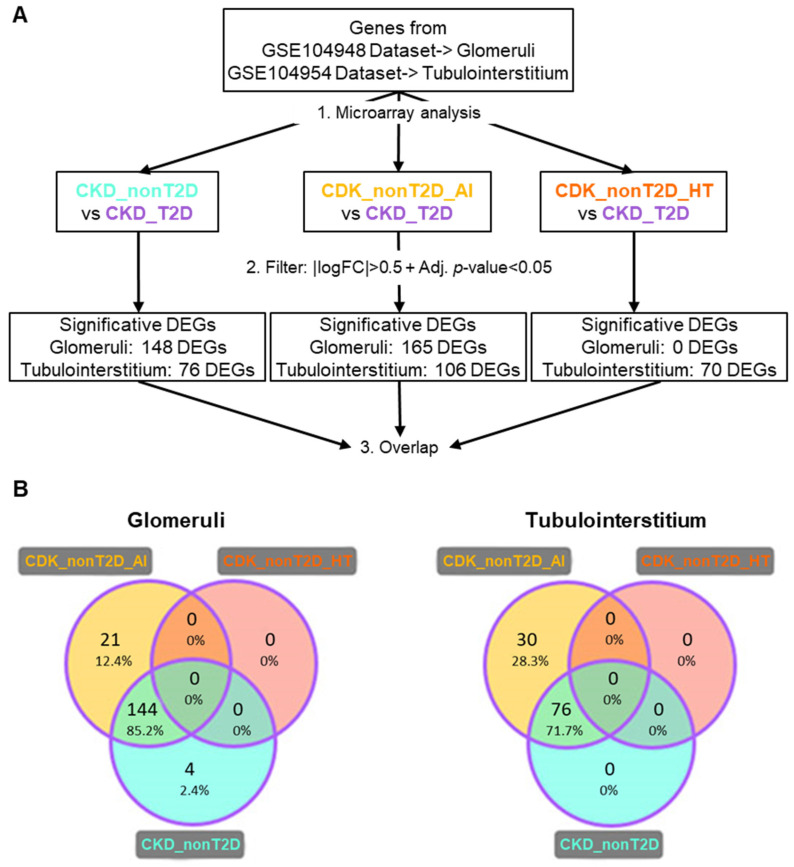
DEGs from direct comparison between the CKD_T2D vs. CKD_nonT2D cohort/subcohorts. (**A**) Description of the path followed during the analysis. Overlapping DEGs are defined as those that show an overlap between CKD_T2D and CKD_nonT2D or any of its subcohorts. (**B**) Venn diagrams for the results of glomeruli and tubulointerstitium direct comparison with CKD_T2D (represented by purple edges). DEGs: Differentially expressed genes; CKD_T2D: chronic kidney disease cohort associated with type 2 diabetes mellitus; CKD-nonT2D: chronic kidney disease cohort not associated with type 2 diabetes; CKD_nonT2D_AI: autoimmune chronic kidney disease; CKD_nonT2D_HT: hypertension CKD. To aid interpretation, different colors were used to distinguish study cohorts: ice blue for CKD_nonT2D, mauve for CKD_nonT2D, topaz for CKD_nonT2D, and tangerine for CKD_nonT2D.

Comparisons were made between the non-diabetic cohort and its subcohorts and the diabetic cohort, as described in Figure 1B, to highlight differences between pathologies. DEGs identified with this strategy are referred to as “direct” DEGs and the results obtained are presented in Figure 3. The results showed no significant direct DEGs, in hypertension CKD compared with diabetic CKD. In contrast, most of the direct DEGs detected for the diabetic cohort coincide with those detected for the autoimmune CKD subcohort. The direct DEGs of autoimmune CKD and non-diabetic CKD were merged, constituting the “direct DEGs” group, which included 169 DEGs for glomeruli and 106 DEGs for tubulointerstitium.

#### 2.2.3. Expression Changes Across CKD Subtypes

In order to compare more in-depth the differences between CKD cohorts/subcohorts, we selected (A) those common DEGs in diabetic and non-diabetic patients in comparison to controls, overlapping in the two conditions (“overlapping DEGs”, see VENN diagram in Figure 2B) that were also direct DEGs (as defined in Section 2.2.2); and (B) those DEGs not shared by diabetic and non-diabetic patients in comparison to controls (“non-overlapping DEGs”, see VENN diagram in Figure 2B) that were also direct DEGs. In the latter group, we identified a small subset showing differential expression in both CKD_T2D and CKD_nonT2D patients relative to controls, but in opposite directions. These were defined as inverse DEGs. For example, a gene may be upregulated in CKD_T2D while being downregulated in CKD_nonT2D_HT or CKD_nonT2D_AI. Such inverse patterns may reflect divergent molecular responses related to disease etiology—such as metabolic stress in T2D versus immune or hemodynamic factors in autoimmune or hypertensive CKD. The identification of these genes may offer valuable insight into etiology-specific mechanisms and highlight potential biomarkers of disease divergence within the broader CKD spectrum.

The selection process described in (A) yielded 56 genes for glomeruli and 52 for tubulointerstitium (overlapping; direct DEGs), and selection B, 83 direct and 18 inverse genes for glomeruli, and 48 direct and 4 inverse genes for tubulointerstitium (non-overlapping; direct or inverse DEGs).

**Figure 4:**
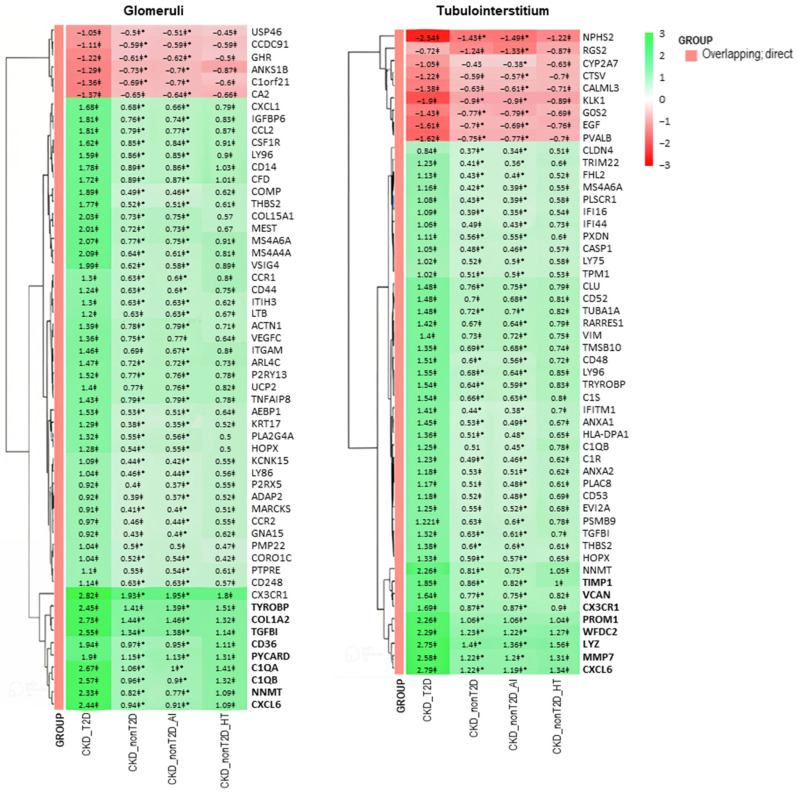
Clustered heatmap of glomerular and tubulointerstitial overlapping genes between CKD and control samples. The heatmap displays logFC values from the “CKD cohort vs. control” comparison. The color scale ranges from red (downregulation) to green (upregulation). Values marked with a double cross (‡) are statistically significant in the “cohort vs. control” comparison (adjusted *p*-value < 0.05). Asterisks (*) denote values that are also significant in the direct comparison between CKD-T2D and CKD-nonT2D. Genes in bold belong to a cluster presenting a higher logFC change in CKD-T2D vs. controls. Hierarchical clustering was applied to both genes and samples. CKD_T2D: chronic kidney disease cohort associated with type 2 diabetes mellitus; CKD-nonT2D: chronic kidney disease cohort not associated with type 2 diabetes; CKD_nonT2D_AI: autoimmune chronic kidney disease; CKD_nonT2D_HT: hypertension CKD.

**Figure 5:**
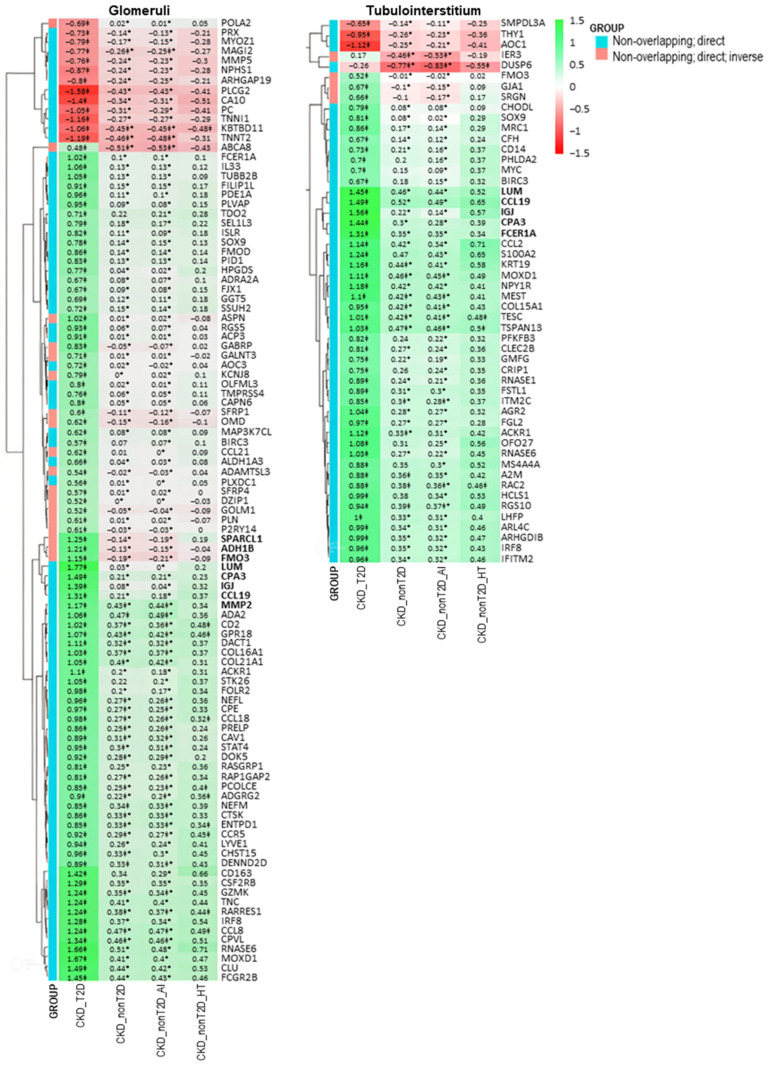
Clustered heatmap of glomerular and tubulointerstitial non-overlapping genes identified in the direct comparison between CKD-T2D and CKD-nonT2D patients. The heatmap displays logFC values from this comparison. The color scale ranges from red (downregulation) to green (upregulation), indicating the direction and magnitude of gene expression change. Genes are classified into two categories (as shown in the GROUP legend): blue indicates “Non-overlapping: direct” genes (differentially expressed only in one population), while red indicates “Non-overlapping: direct; inverse” genes—i.e., genes that show opposite regulation direction between CKD-T2D and CKD-nonT2D when compared to controls. Values marked with a double cross (‡) are statistically significant in the “CKD cohort vs. control” comparison (adjusted *p*-value < 0.05). Asterisks (*) denote values that are statistically significant in the direct comparison (adjusted *p*-value < 0.05 and |logFC| > 0.5). Genes in bold belong to a cluster presenting a higher logFC change in CKD-T2D vs. controls. Hierarchical clustering was applied to both genes and samples.

To better visualize changes of expression patterns of those selected DEGs, clustered heatmaps were generated (Figure 4 and Figure 5) and allowed identifying DEGs with consistent behavior in glomeruli and tubulointerstitium, highlighted in bold in the heatmaps.

**Figure 6:**
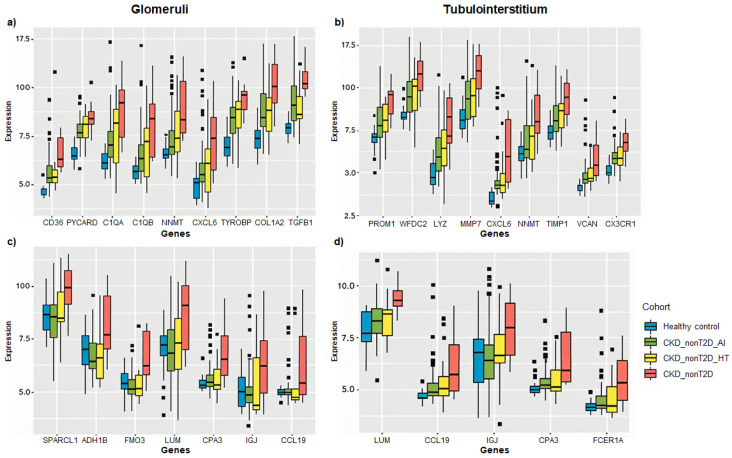
Box plots showing the expression of selected DEGs across CKD subtypes relative to healthy controls. Genes were selected for their consistent behavior in both renal compartments (glomeruli and tubulointerstitium) and for presenting some of the highest log fold change (logFC) values across comparisons. Panels (**a**,**b**) display overlapping direct genes, while panels (**c**,**d**) present non-overlapping direct and inverse genes. Expression is represented as logFC values for each comparison between CKD subgroups (CKD_T2D, CKD_nonT2D_AI, CKD_nonT2D_HT) and controls. Panels a and c correspond to glomerular data, and panels b and d to tubulointerstitial data. Data were obtained from chip definition files of GSE104948 (glomeruli) and GSE104954 (tubulointerstitium).

These genes with consistent behaviors between the studied cohorts are shown in Figure 6. Specifically, overlapping and direct genes such as *Cxcl6* and *Nnmt* and non-overlapping and direct genes such as *Ccl19*, *Cpa3*, *Igj*, and *Lum* are observed both in glomeruli and tubulointerstitium, reflecting common expression patterns. Furthermore, changes in gene expression in the CKD-T2D cohort are more pronounced than those in the overall CKD cohort, highlighting significant differences in the molecular response to type 2 diabetes within the context of the renal disease. On the other hand, hypertension-CKD shows greater similarity to CKD_T2D than autoimmune-CKD, indicating a possible pathological alignment between hypertension and diabetic complications in chronic kidney disease.

### 2.3. Enriched Pathways in Chronic Kidney Diseases

**Figure 7:**
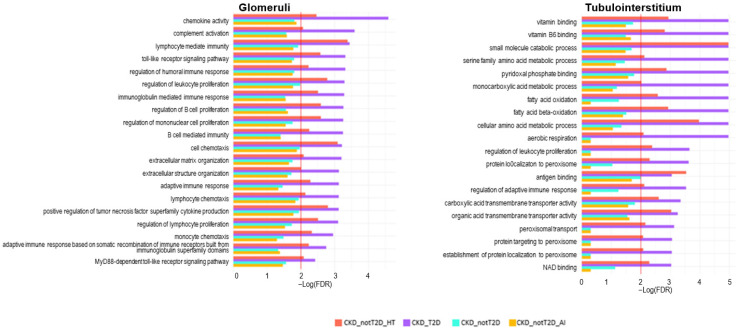
Significantly enriched processes in CKD_T2D but not in CKD_nonT2D. The figure illustrates the results of gene set enrichment analysis (GSEA), depicting significantly enriched biological processes in glomerular and tubulointerstitial compartments across four CKD subtypes: hypertensive CKD (CKD_nonT2D_HT), diabetic CKD (CKD_T2D), overall non-diabetic CKD (CKD_nonT2D), and autoimmune CKD (CKD_nonT2D_AI). Bars represent enrichment significance as –log(FDR), with higher values indicating greater statistical significance. The red line shows the proportion of null hypotheses in the data.

**Figure 8:**
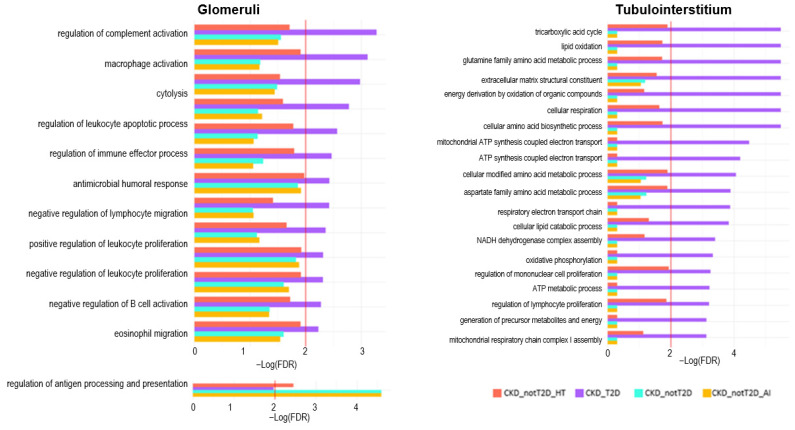
Significantly enriched processes in CKD_T2D and hypertension-CKD in glomeruli and tubulointerstitium. Depicts the results of gene set enrichment analysis (GSEA) in the glomerular and tubulointerstitial compartments across CKD cohorts. The enrichment significance is represented as –log(FDR), with higher values indicating stronger enrichment. The red line shows the proportion of null hypotheses in the data.

A GSEA analysis was performed to explore biological differences between the cohorts. Thus, GSEA analysis was carried out based on the information obtained from the differential expression analysis for the biological interpretation of the results. Genes ranked by logFC were used as an input for each cohort/subcohort in order to obtain sets up or downregulated for each one of them. Only sets with an FDR q-value < 0.01 and a set size between 10 and 500 genes/proteins were selected. The results of these analyses are provided in a tissue-specific table (Supplementary Table S1), where all significant gene/protein sets that are over- or underexpressed are listed. A total of 13 gene sets were identified as significantly enriched in diabetic patients but not in the non-diabetic in glomeruli and 117 for tubulointerstitium. Only one gene set in glomeruli and none in tubulointerstitium were significantly enriched in non-diabetic patients and their subcohorts and not in diabetic ones. A total of 35 gene sets for glomeruli and 77 for tubulointerstitium were significantly enriched only in diabetic and hypertension cohorts. From these, the 20 gene sets with a lower FDR per comparison were represented in Figure 7 and Figure 8.

In order to provide further insights on the implications of the results, these sets were classified into functional groups, according to a bibliographic review on kidney disease and T2D pathophysiology. Focusing on enriched processes in the CKD_T2D but not in the CKD_nonT2D cohorts and their subcohorts (Figure 7), we found that in general, glomeruli have more immune-mediated enriched processes and tubulointerstitium have more metabolism-associated enriched processes. In the glomerular compartment, enriched pathways were predominantly immune-related across all subtypes. Notably, diabetic CKD (purple) displayed the highest enrichment in processes such as regulation of complement activation, leukocyte apoptotic process, macrophage activation, and cytolysis, highlighting robust immune and inflammatory activity. This is consistent with the known role of complement and immune dysregulation in diabetic nephropathy. Interestingly, regulation of antigen processing and presentation was uniquely enriched in non-diabetic subtypes, especially autoimmune CKD, suggesting distinct immunological mechanisms in glomerular injury across etiologies. In contrast, the tubulointerstitial compartment revealed a predominance of metabolic processes. Diabetic CKD again showed the most significant enrichment in pathways related to mitochondrial function, including the TCA cycle, oxidative phosphorylation, NADH dehydrogenase complex assembly, and fatty acid oxidation, consistent with metabolic reprogramming. Non-diabetic subtypes, particularly autoimmune CKD, showed less enrichment in these pathways. Interestingly, extracellular matrix structural constituent were enriched in diabetic CKD, further supporting the fibrotic remodeling phenotype in the tubulointerstitium of these patients.

Focusing on processes enriched in diabetic CKD and also in hypertension-CKD (Figure 8), we found similar results as before, more immune-related in glomeruli, where terms such as chemokine activity, complement activation, lymphocyte-mediated immunity, and toll-like receptor signalling pathways are significantly enriched in CKD_T2D, indicating heightened innate and adaptive immune responses in the glomerular compartment of diabetic patients. In contrast, the tubulointerstitial compartment showed a stronger enrichment of metabolic and transport-related pathways. In particular, CKD_T2D exhibited activation of pathways involved in vitamin metabolism (e.g., vitamin B6 binding, pyridoxal phosphate binding), fatty acid oxidation, and mitochondrial/peroxisomal function (e.g., aerobic respiration, protein targeting/localization to peroxisomes, peroxisomal transport). Beyond general metabolic categories, some of these processes converge on well-defined regulatory axes such as PPAR signaling, which orchestrates lipid metabolism and mitochondrial β-oxidation, and mTOR signaling, a central pathway in nutrient sensing, cellular growth, and metabolic homeostasis. The enrichment of these pathways in CKD_T2D suggests a more profound metabolic remodeling in the tubulointerstitium under diabetic conditions, integrating both basal bioenergetic processes and higher-order regulatory responses, potentially contributing to disease-specific progression in a compartment-specific manner.

Altogether the GSEA analysis indicates a compartmentalized molecular pattern in CKD subtypes, with most of the pathophysiological changes in the glomeruli related to the overactivation of the immune system. In contrast, in the tubulointerstitium, metabolic changes may contribute to a greater extent to disease progression in CKD_T2D (and to some extent in CKD_nonT2D_HT) compared to CKD_nonT2D_AI.

## 3. Discussion

This integrative transcriptomic analysis provides a comparative landscape of gene expression alterations in CKD_T2D and CKD_nonT2D, revealing both overlapping and divergent molecular signatures across kidney tissues. While overall expression changes in CKD_T2D and CKD_nonT2D are similar when compared to healthy controls, several key differences emerged upon direct comparison between diabetic and nondiabetic samples. First, diabetic samples exhibited a broader set of DEGs and higher logFC values compared to non-diabetics, indicating a more extensive molecular disturbance in diabetic kidney disease, where roughly half of DEGs are not present in CKD_nonT2D, suggesting that kidneys from the diabetic populations also have other changes not present in the non-diabetic cohort. This suggests that hyperglycemia, its associated metabolic dysfunction, and chronic metabolic stress [10,11], together, potentiate transcriptomic changes beyond those observed in autoimmune or hypertensive CKD. Interestingly, our findings also highlight a notable similarity between CKD_T2D and CKD_nonT2D_HT, particularly in the enrichment of metabolic pathways in the tubulointerstitial compartment. This may reflect shared pathophysiological mechanisms between diabetic and hypertensive CKD. Both conditions are characterized by systemic and intrarenal endothelial dysfunction, leading to impaired vasodilation, capillary rarefaction, and altered glomerular hemodynamics [12]. Additionally, oxidative stress has been widely recognized as a common pathogenic denominator in T2D and hypertension, promoting mitochondrial dysfunction, chronic inflammation, and progressive fibrosis [13,14]. In this context, the observed activation of mitochondrial and peroxisomal pathways in both CKD_T2D and CKD_nonT2D_HT may reflect a shared compensatory response to heightened oxidative and metabolic stress. These similarities suggest that overlapping molecular responses—particularly in tubular compartments—might contribute to disease progression through converging mechanisms. This potential convergence suggests that therapeutic strategies targeting mitochondrial resilience, redox balance, or endothelial function may be beneficial in both CKD subtypes.

Within the non-diabetic CKD cohort, hypertensive and autoimmune etiologies presented distinct expression profiles. Interestingly, CKD_nonT2D_HT showed greater similarity to CKD_T2D than the autoimmune counterpart, consistent with clinical evidence linking systemic and intraglomerular hypertension to diabetic nephropathy. This may reflect shared activation of pathways such as the renin–angiotensin–aldosterone system (RAAS), a common driver of glomerular injury in both conditions [15,16]. We identified and highlighted a selection of genes with significant differential expression across tissues, particularly those that demonstrate the most pronounced quantitative shifts between disease cohorts and controls. These genes, whether overlapping or non-overlapping differentially expressed genes (DEGs) with respect to the control cohort, were clustered based on their tissue-specific or shared expression patterns. Their biological roles were further categorized into pathways related to extracellular matrix (ECM) remodeling, immune and inflammatory responses, diabetes-related alterations, complement system activation, apoptosis, and emerging molecular candidates of unclear function in CKD or T2D.

A substantial subset of DEGs was associated with ECM remodeling, a hallmark of chronic kidney disease (CKD) progression. In glomerular tissue, *Tgfb1* was markedly upregulated in response to hyperglycemic stimuli [17], consistent with its known role in promoting type I collagen synthesis. This was corroborated by the concurrent overexpression of *Col1a2*, a principal ECM component, which underscores the fibrotic response initiated by elevated glucose levels [18]. Within the tubulointerstitial compartment, we observed increased expression of *Timp1*, a known inhibitor of metalloproteinases, alongside *Vcan*, which contributes to ECM-cell interactions and intercellular signaling [19]. Additional upregulated genes such as *Wfdc2* and *Mmp7* further support the notion of active ECM deposition in this compartment [20,21]. Notably, *Cxcl6*, *Ccl19*, and *Lum* were found to be upregulated in both glomeruli and tubulointerstitium, suggesting a shared fibrotic axis [22,23]. These genes are functionally linked to TGF-β1 signaling and likely key mediators in fibrosis development. Interestingly, *Nnmt*, which also appeared in both tissues, may represent a compensatory anti-fibrotic mechanism [24], as previously proposed.

Beyond fibrotic remodeling, a number of DEGs were implicated in immune regulation and inflammatory signaling. Within the glomeruli, *Tyrobp* emerged as a prominent gene, previously associated with immune function and autoimmune CKD, based on reanalysis of public datasets (GSE93798, GSE37460) [25]. In the tubulointerstitial tissue, *Cx3cr1* was notably enriched, consistent with its roles in immune surveillance, leukocyte adhesion, and chemotaxis [26]. *Cxcl6* and *Ccl19*, beyond their involvement in ECM remodeling, also possess proinflammatory properties [27,28] and were differentially expressed in both compartments, reflecting the complex interplay between inflammation and fibrosis.

We also identified DEGs with established links to diabetes-related processes. In the glomeruli, the Adh1b gene has been found to be overexpressed in human perirenal adipose tissue-derived mesenchymal stem cells [29]. This is the first report demonstrating that Adh1b is also upregulated in glomeruli, indicating a direct association with insulin resistance and suggesting that metabolic stress may directly influence glomerular gene expression profiles. Conversely, in the tubulointerstitial region, Lyz was found to be upregulated. A previous study using in vitro HK-2 cells showed that human recombinant Lyz downregulates interleukin-6 (IL-6) production induced by advanced glycation end products (AGEs). This effect may represent a protective mechanism that counteracts AGE-induced inflammation [30].

A pronounced overactivation of the complement system was observed predominantly in glomerular tissue. Genes such as *C1qa* and *C1qb* [31] were significantly overexpressed in diabetic CKD compared to non-diabetic CKD, displaying notable log fold change (logFC < −1.6). *Igj*, another complement-associated gene, was also detected in both compartments, underscoring a systemic activation of this pathway, particularly in the diabetic context.

Interestingly, genes typically associated with tubular apoptosis were differentially expressed in the glomerular compartment. *Cd36*, known to mediate AGE-BSA-induced apoptosis [32], and *Pycard* (also known as *Asc*), a key inflammasome component [33], were both upregulated, suggesting possible cross-talk between glomerular and tubular compartments. *Pycard* also connects mechanistically to SGLT2 pathways; in fact, dual DPP4- and SGLT2-inhibitor therapies have been shown to suppress NLRP3/ASC inflammasome activation [34]. As inflammasome activation is closely tied to endoplasmic reticulum (ER) stress, and ER stress is a known driver of tubular apoptosis [35,36], this observation may highlight an underappreciated axis of intercompartmental communication in CKD pathogenesis.

Lastly, we identified a set of genes whose roles in CKD or T2D have not yet been fully characterized, although preliminary data suggest potential involvement. These include *Sparcl1* and *Fmo3* in glomeruli, *Prom1* and *Fcer1a* in the tubulointerstitium, and *Cpa3*, which was upregulated in both tissues. The functions of these genes remain to be elucidated but may represent novel biomarkers or therapeutic targets [37].

In summary, the DEGs expressed in both glomerular and tubulointerstitial tissues largely converge on fibrotic and proinflammatory pathways, with TGF-β1-mediated ECM remodeling and inflammation representing core mechanisms. This aligns with current knowledge that positions diabetes as a driver of chronic inflammation and fibrosis, ultimately exacerbating renal damage [38].

Our findings underscore the value of comparative transcriptomics in unraveling the shared and tissue-specific molecular landscapes underpinning diabetic and non-diabetic CKD. A key strength of this study is the use of kidney tissue datasets, which allow for more accurate cohort classification and mitigate potential misclassification bias common in population-based studies without histological confirmation.

Gene set enrichment analysis (GSEA) further complemented these transcriptomic findings by elucidating the broader functional implications of gene expression changes across tissues and disease subtypes. When comparing CKD and CKD-T2D cohorts to controls, both displayed a similar landscape of altered biological processes, emphasizing common pathological mechanisms driving renal dysfunction. However, direct comparison between CKD and CKD-T2D revealed more pronounced transcriptional divergence in the tubulointerstitial compartment than in the glomeruli, suggesting that tubulointerstitial tissue may serve as a more sensitive indicator of diabetes-specific molecular alterations [39].

In terms of enriched biological functions, consistent with individual gene-level findings, processes related to ECM remodeling and immune system activation were markedly enriched in CKD-T2D relative to CKD across both renal compartments. Complement activation remained a glomerulus-specific hallmark, reflecting its strong association with immune complex-mediated damage in this region. Interestingly, metabolic disturbances—including those associated with metabolic acidosis, lipid remodeling, and dyslipidemia—were found to be uniquely enriched in the tubulointerstitium of CKD-T2D patients. While prior studies have implicated these pathways in both diabetic and non-diabetic CKD [40,41,42,43], our data suggest a higher prevalence or intensity of these alterations in the diabetic context, particularly in the tubular environment. These findings emphasize the multifaceted impact of diabetes on renal physiology, extending beyond fibrosis and inflammation to include significant metabolic reprogramming, especially at the level of the tubulointerstitium.

## 4. Materials and Methods

### 4.1. Data Selection

#### 4.1.1. Databases Used and Search Strategies

High-throughput data compilation was carried out in two different database repositories, Gene Expression Omnibus (GEO) [44] and ArrayExpress [45]. In both databases, a search for gene expression data was performed for the medical condition “chronic kidney disease” (including its MeSH and Malacards synonyms). In addition, the same search strategy was used for ‘diabetic nephropathy,’ incorporating the term of this medical condition along with its MeSH and Malacards database synonyms. Searches were also restricted for Homo sapiens (organism).

#### 4.1.2. Inclusion and Exclusion Criteria

For this study, only Homo sapiens gene expression data obtained using high-precision techniques such as array expression profiling and high-throughput sequencing were included. Single-cell RNA-seq studies were excluded due to their non-comparable results with the mRNAseq technique. Studies that analyzed renal transplant samples and cases of autosomal dominant or autosomal recessive polycystic kidney disease, lupus nephritis, or antineutrophil cytoplasmic antibody (ANCA)-associated vasculitis were also excluded. Additionally, studies with a sample size for any cohorts of interest less than ten were discarded. Finally, an exhaustive review of the scientific literature associated with the included studies was carried out.

#### 4.1.3. Description and Selections of the Cohorts

This study established various cohorts and subcohorts based on the etiology of chronic kidney disease: CKD cohort associated with type 2 diabetes (CKD_T2D) and the non-diabetic associated (CKD_nonT2D), which was further divided into the autoimmune subcohort (Autoimmune CKD, hereafter defined as CKD_nonT2D_AI), which includes subpathologies, such as focal and segmental glomerulosclerosis, IgA nephropathy, thin membrane disease, minimal change disease, and membranous glomerulonephritis, and the hypertensive CKD subcohort (Hypertension CKD, hereafter defined as CKD_nonT2D_HT). The datasets were selected based on the specific tissues found in the kidneys (glomerulus and/or tubuli). More information will be found in Supplementary Methods.

### 4.2. Data Treatment and Analysis

Raw gene expression data were quantile normalized and batch-corrected using the COMBAT method [46]. Differential expression analyses were carried out using the Limma package from R version 3.46.0 (R Foundation for Statistical Computing, Vienna, Austria). For identifying differentially expressed genes, thresholds of Adj. *p*-value < 0.05 and an absolute log fold change (|logFC|) > 0.5 were established. The Benjamini–Hochberg approach was used as a multi-test correction for each database [47]. Gene Set Enrichment Analysis (GSEAs) was performed using the list of differentially expressed genes/proteins ranked by their logFC. The number of permutations in the GSEA was set to 1000. The GO_BIOLOGICAL_PROCESS and GO_MOLECULAR_FUNCTION databases [48] were used for the analysis. The Benjamini–Hochberg approach was used as a multi-test correction for each database, and a False Discovery Rate (FDR) q-value < 0.01 was considered significant. Moreover, only gene/protein sets with a set size between 10 and 500 genes/proteins were selected in order to discard unspecific or too general results [49].

## 5. Conclusions

In conclusion, our findings provide a comprehensive transcriptomic overview of diabetic and non-diabetic CKD, highlighting both shared and distinct molecular pathways across renal compartments. Diabetic CKD exhibited a broader spectrum of transcriptomic alterations, particularly in the tubulointerstitial compartment, where metabolic and fibrotic processes were strongly enriched. In contrast, immune-related changes predominated in glomeruli, especially via complement activation. The comparative analysis also revealed that hypertensive CKD shares greater molecular resemblance with diabetic CKD than the autoimmune subtype. These results support the hypothesis that diabetes triggers a multifactorial and compartment-specific molecular response that amplifies inflammation, fibrosis, and metabolic stress. Ultimately, our study underscores the utility of transcriptomic profiling to dissect the complex pathogenesis of CKD and advocate for precision medicine strategies tailored to disease etiology and tissue-specific mechanisms.

## Data Availability

The original contributions presented in this study are included in the article. Further inquiries can be directed to the corresponding authors.

## Supplementary Materials

The following supporting information can be downloaded at: https://www.mdpi.com/article/10.3390/ijms26157421/s1. Reference [50] is cited in the Supplementary Materials.

## Abbreviations

The following abbreviations are used in this manuscript:

AGEs	advanced glycation end-products
AI	autoimmune
ANCA	anti-neutrophil cytoplasm antibodies
BSA	bovine serum albumin
CKD	chronic kidney disease
DEGs	differentially expressed genes
DPP4	dipeptidyl peptidase 4
ECM	extracellular matrix
FDR	false discovery rate
GEO	gene expression omnibus
GSEA	gene set enrichment analysis
GWAS	genome-wide association studies
HT	hypertensive
logFC	log fold change
NADH	nicotinamide adenine dinucleotide
SGLT2	sodium–glucose cotransporter 2
T2D	type 2 diabetes mellitus
TCA	tricarboxylic acid
