# Supplementary material for: Identification of Differentially Expressed Genes and Pathways in Non-Diabetic CKD and Diabetic CKD by Integrated Human Transcriptomic Bioinformatics Analysis
Source: Int J Mol Sci. 2025 Aug 1;26(15):7421. doi: 10.3390/ijms26157421 (PMC12347806; doi:10.3390/ijms26157421)
Supplement: Supplementary file 1 [file ijms-26-07421-s001.zip › Supplementary Figure S1.docx]

**Supplementary Figure S1:**


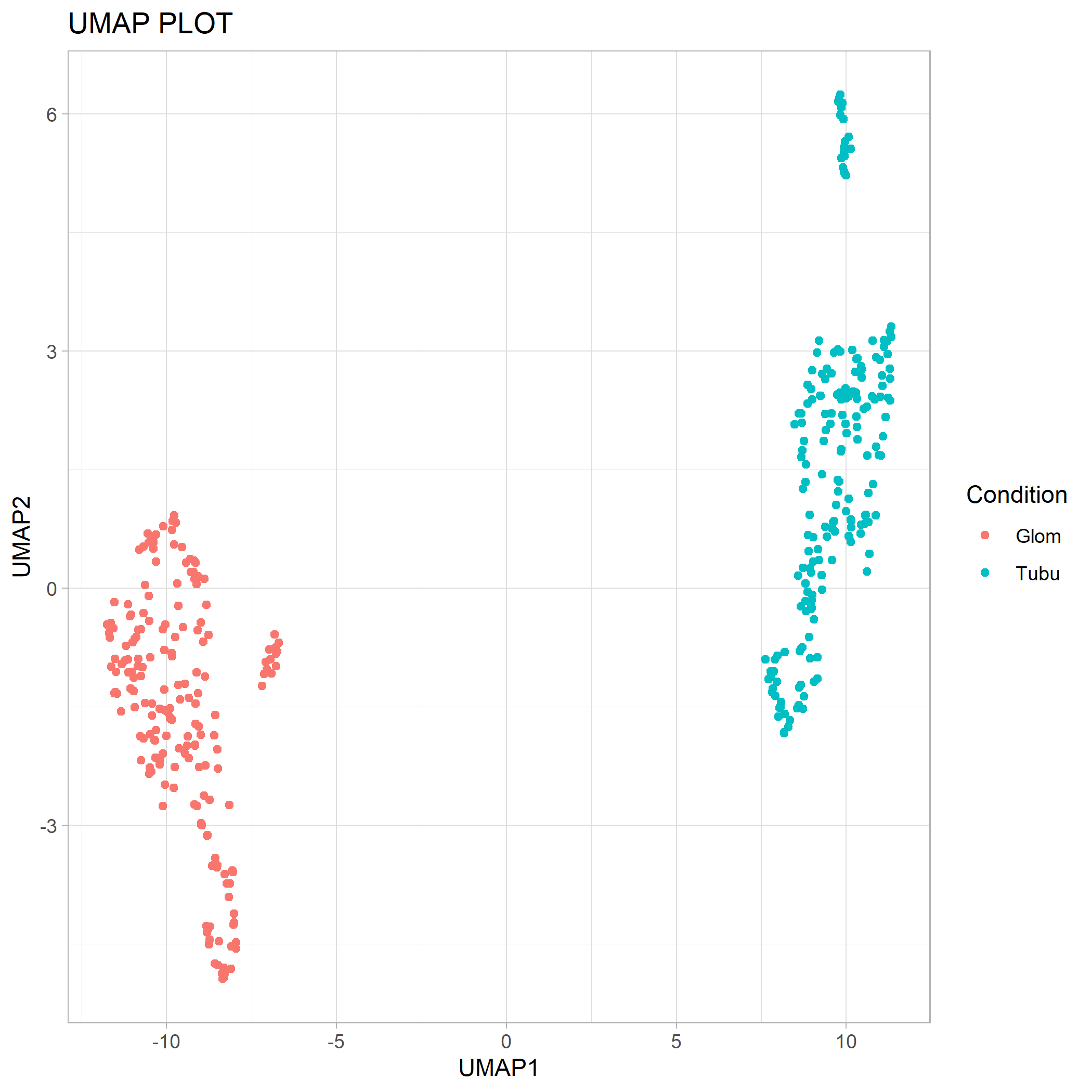


**Supplementary Figure S1**: Metadata clustering of selected datasets. Samples clustered according to tissue. As an example, figure illustrates these differences for the datasets

GSE104954 (tubulointerstitium) and GSE104948 (glomeruli). Same observation was done for the others datasets.
