# Supplementary material for: Identification of Differentially Expressed Genes and Pathways in Non-Diabetic CKD and Diabetic CKD by Integrated Human Transcriptomic Bioinformatics Analysis
Source: Int J Mol Sci. 2025 Aug 1;26(15):7421. doi: 10.3390/ijms26157421 (PMC12347806; doi:10.3390/ijms26157421)
Supplement: Supplementary file 1 [file ijms-26-07421-s001.zip › Supplementary Figure S2.docx]

**Supplementary Figure S2:**


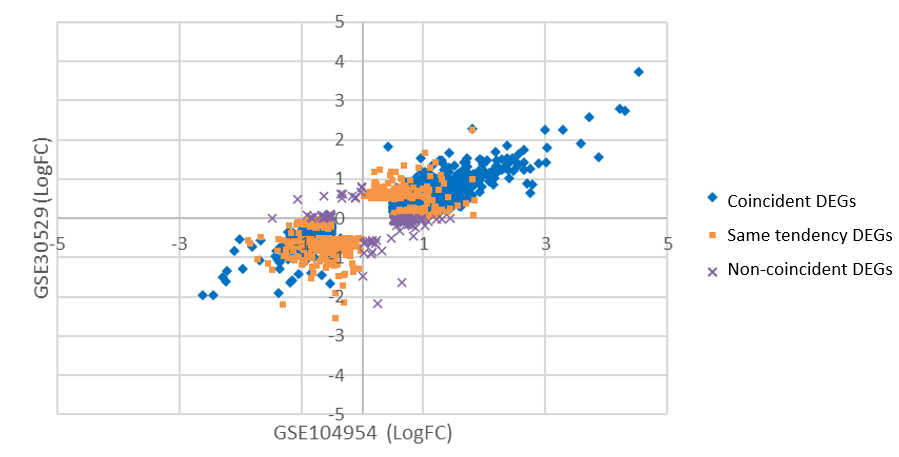


Supplementary Figure S2: Cross-checking of CKD-T2D vs control comparison by using GSE30529 dataset. DEGs distribution of GSE30529 (validator) AND GSE104954 (dataset of main analysis). Blue squares represent the 373 coincident DEGs; orange squares represent those DEGs with the same tendency of logFC but not significative (adj. p-value) in at least one of the datasets; purple crosses represent those inverse DEGs between both datasets. GSE30529 dataset contains 10 samples CKD-T2D and 12 LD from tubulointerstitium biopsies.
