# Supplementary material for: Identification of Differentially Expressed Genes and Pathways in Non-Diabetic CKD and Diabetic CKD by Integrated Human Transcriptomic Bioinformatics Analysis
Source: Int J Mol Sci. 2025 Aug 1;26(15):7421. doi: 10.3390/ijms26157421 (PMC12347806; doi:10.3390/ijms26157421)
Supplement: Supplementary file 1 [file ijms-26-07421-s001.zip › Supplementary Methods.docx]

**Supplementary Methods:**

**1:** **High-throughput data (HTD) compilation** was carried out in two different database repositories, Gene Expression Omnibus (GEO) [1] and ArrayExpress [2].

First, a search for gene expression data was performed in the GEO database (https://www.ncbi.nlm.nih.gov/geo/) for the medical condition CKD. In order to design the following search, MeSH synonyms (https://www.ncbi.nlm.nih.gov/mesh/) and Malacards database synonyms (https://www.malacards.org) for the term “chronic kidney disease “were used. The purpose of it was to englobe as much significant results as possible. Search was also restricted for Homo sapiens (organism) and for specific techniques (as shown in the search string below). 77 results were obtained for this search in GEO repository.

**Chronic kidney disease**: ("renal insufficiency, chronic"[MeSH Terms] OR "chronic kidney disease"[All Fields] OR "chronic kidney diseases"[All Fields] OR "CKD"[All Fields] OR "chronic renal insufficiency"[All Fields] OR "kidney failure, chronic"[MeSH Terms] OR "chronic renal disease"[All Fields] OR "chronic renal disease"[All Fields] OR "chronic kidney failure"[All Fields] OR "chronic renal.

For the medical condition CKD-T2D, same search as the previous one was carried out adding the term “diabetic nephropathy” together with its MeSH and Malacards database synonyms. Search string is shown below. 18 results were obtained for this search in GEO repository.

**Chronic kidney disease and T2D**: ("renal insufficiency, chronic"[MeSH Terms] OR "chronic kidney disease"[All Fields] OR "chronic kidney diseases"[All Fields] OR "CKD"[All Fields] OR "chronic renal insufficiency"[All Fields] OR "kidney failure, chronic"[MeSH Terms] OR "chronic renal disease"[All Fields] OR "chronic renal disease"[All Fields] OR "chronic kidney failure"[All Fields] OR "chronic renal failure"[All Fields]) AND "Homo sapiens"[Organism] AND ("diabetes mellitus, type 2"[MeSH Terms] OR "type 2 diabetes"[All Fields] OR "diabetic nephropathies"[MeSH Terms] OR "diabetic nephropathy"[All Fields] OR "diabetic nephropathies"[All Fields] OR "diabetic kidney disease"[All Fields] OR "Nodular Glomerulosclerosis"[All Fields]) AND ("Expression profiling by array"[Filter] OR "Expression profiling by high throughput sequencing"[Filter] OR "Protein profiling by Mass Spec"[Filter] OR "Protein profiling by protein array"[Filter]) AND "gse"[Filter]

Afterwards, ArrayExpress database (https://www.ebi.ac.uk/arrayexpress/) was explored in order to find further HTD regarding CKD and CKD+T2D. For the medical condition CKD, “chronic kidney disease” MeSH and Malacards database synonyms were used. The results obtained were then filtered by Homo sapiens and RNA assay. Search string is shown below. 54 results were obtained from this search.

**Chronic kidney disease**: ("chronic kidney disease" OR "chronic renal insufficiency" OR "chronic renal insufficiencies" OR "chronic kidney insufficiency" OR "chronic kidney insufficiencies" OR "chronic kidney diseases" OR "chronic renal disease" OR " chronic renal diseases" OR "chronic kidney failure" OR "chronic renal failure")

Filtered by organism Homo sapiens, experiment type "rna assay"

For the medical condition CKD-T2D, same search as the previous one was carried out adding the term “diabetic nephropathy” together with its MeSH and Malacards database synonyms. The results obtained were then filtered by Homo sapiens and RNA assay. Search string is shown below. Search string is shown below. 25 results were obtained for this search.

**Chronic kidney disease and T2D**: ("chronic kidney disease" OR "chronic renal insufficiency" OR "chronic renal insufficiencies" OR "chronic kidney insufficiency" OR "chronic kidney insufficiencies" OR "chronic kidney diseases" OR "chronic renal disease" OR "chronic renal diseases" OR "chronic renal disease" OR "chronic renal diseases" OR "chronic kidney failure" OR "chronic renal failure") AND ("diabetes mellitus, type 2" OR "type 2 diabetes" OR "diabetic nephropathies" OR "diabetic nephropathy" OR "diabetic nephropathies" OR "diabetic kidney disease" OR "Diabetic Glomerulosclerosis" OR "Intracapillary Glomerulosclerosis" OR "Nodular Glomerulosclerosis" OR "Kimmelstiel-Wilson Syndrome" OR "Kimmelstiel Wilson Syndrome" OR "Kimmelstiel-Wilson Disease" OR "Kimmelstiel Wilson Disease")

Filtered by organism Homo sapiens, experiment type "rna assay"

**2: Exclusion/Inclusion Criteria**

In order to select those accurate and most representative datasets, a first selection of gene expression studies performed in *Homo sapiens* were accepted. Subsequently, the following indications were considered to finally select the most useful datasets to represent both medical conditions:

It is well known that chronic kidney disease can be caused by several pathologies, directly related to the severity of CKD. In order to include these subpathologies (such as membranous nephropathy, focal segmental glomerulosclerosis, minimal change disease, among others), eGFR (estimated glomerular filtration rate) data was reviewed for each of them. As in DAPA-CKD study [3] exclusion criteria, only those samples with an eGFR between 25 and 75 mL/min/1.73m^2^ were considered. Focal segmental glomerulosclerosis was the only subpathology that matched eGFR restriction thus it was considered for the CKD cohort. Stages IV and V of CKD (eGFR<29 mL/min/1.73m^2^) as well as end-stage renal disease (ESRD; eGFR<15 mL/min/1.73m^2^) were automatically excluded. As agreed with the client, hypertension and autoimmune-associated CKD were systematically included in the CKD cohort.

Additionally, kidney allografts (kidney transplantation), autosomal dominant or autosomal recessive polycystic kidney disease, lupus nephritis or anti-neutrophil cytoplasmic antibody (ANCA)-associated vasculitis samples were excluded following the DAPA-CKD study exclusion criteria.

Experiments carried out using single cell-RNAseq technique were excluded as they were considered non-comparable with mRNAseq technique.

Studies with sample population size (N) lower than 10 were excluded.

An exhaustive review of the scientific literature associated to the retrieved studies was carried out. From the literature relevant information was extracted for this phase of the project and the following ones.

**References:**

1. Edgar, R.; Domrachev, M.; Lash, A.E. Gene Expression Omnibus: NCBI Gene Expression and Hybridization Array Data Repository. *Nucleic Acids Res.* **2002**, *30*, 207–210.

2. Athar, A.; Füllgrabe, A.; George, N.; Iqbal, H.; Huerta, L.; Ali, A.; Snow, C.; Fonseca, N.A.; Petryszak, R.; Papatheodorou, I.; et al. ArrayExpress Update - from Bulk to Single-Cell Expression Data. *Nucleic Acids Res.* **2019**, *47*, D711–D715.

3. Heerspink, H.J.L.; Stefánsson, B. V.; Correa-Rotter, R.; Chertow, G.M.; Greene, T.; Hou, F.-F.; Mann, J.F.E.; McMurray, J.J.V.; Lindberg, M.; Rossing, P.; et al. Dapagliflozin in Patients with Chronic Kidney Disease. *N. Engl. J. Med.* **2020**, *383*, 1436–1446.
