# Supplementary material for: Identification of Differentially Expressed Genes and Pathways in Non-Diabetic CKD and Diabetic CKD by Integrated Human Transcriptomic Bioinformatics Analysis
Source: Int J Mol Sci. 2025 Aug 1;26(15):7421. doi: 10.3390/ijms26157421 (PMC12347806; doi:10.3390/ijms26157421)
Supplement: Supplementary file 1 [file ijms-26-07421-s001.zip › Supplementary Table S1_v2.docx]

**Supplementary Table S1:**

Excel file contains the complete list of differentially expressed genes (DEGs) identified during the transcriptomic analysis. Sheet 1 – Summary: Provides an overview of the file content, including the number of DEGs found per comparison and direct links to the corresponding sheets. Subsequent Sheets: Present DEG data grouped by DEG classification (e.g., Overlapping; Direct, Non-overlapping; Inverse) and by renal tissue (glomeruli or tubulointerstitium). Each row includes log fold change (LogFC) and adjusted p-value from both the “CKD cohort vs control” and the “CKD_T2D vs CKD_nonT2D” comparison. Genes not meeting DEG thresholds (|LogFC| > 0.5 and adjusted p < 0.05) are highlighted in orange.
